# What Are Complex Interventions in Suicide Research? Definitions, Challenges, Opportunities, and the Way Forward

**DOI:** 10.3390/ijerph19148591

**Published:** 2022-07-14

**Authors:** Sadhvi Krishnamoorthy, Sharna Mathieu, Victoria Ross, Gregory Armstrong, Kairi Kõlves

**Affiliations:** 1 Australian Institute for Suicide Research and Prevention, World Health Organization Collaborating Centre for Research and Training in Suicide Prevention, School of Applied Psychology, Griffith University, Brisbane, QLD 4122, Australia; s.mathieu@griffith.edu.au (S.M.); victoria.ross@griffith.edu.au (V.R.); 2 Nossal Institute for Global Health, Melbourne School of Population and Global Health, University of Melbourne, Parkville, VIC 3010, Australia; g.armstrong@unimelb.edu.au

**Keywords:** complex interventions, suicide research, suicide prevention, multilevel, multicomponent, multimodal, systems approach, community-based, definitions, implementation

## Abstract

It has been argued that effective action towards addressing a complex concern such as suicide requires a combination of evidence-based strategies. While these complex public health approaches have recently gained importance, little is known about their characteristics and what contributes to their complexity. The use of interchangeable terms such as *multilevel*, *multicomponent*, *community based*, and inconsistent definitions of these approaches creates confusion around what it is and what it is not. In practice, this disorder is reflected in a substantial variation in the design, implementation, and evaluation of complex approaches in suicide research. While it is impossible to resolve all existing inconsistencies in terminology, this review explores a range of terms and definitions to connote *complex interventions*. It aims to unpack multiple meanings of these terms and their diverse usage in suicide literature. The potential implications of this fluidity and plausible pathways to make sense of this complexity for suicide research are also discussed. With a shared understanding of what constitutes a *complex intervention*, we can expect to see an improved representation of the real-world complexities in our efforts to address suicide. This common language can also contribute toward quality implementation and dissemination and thereby advance our understanding of complex interventions.

## 1. Introduction

Suicidal behavior is complex and determined by several intertwined factors. Contributions of individual as well as population level risk factors highlight the heterogeneity in its etiology. These risk factors further vary in strength and patterns of association across gender, age, culture, location, and individual history [1]. There is now an emerging evidence base for a range of interventions to prevent as well as treat suicidal behavior. For example, it has been found that interventions such as training primary care physicians in depression recognition and treatment, educating youths on depression and suicidal behavior, as well as active outreach to psychiatric patients after discharge help prevent suicide [2]. The effectiveness of interventions across a spectrum of universal, selective, and indicated interventions is variable [2], with each intervention holding its relevance and importance across different contexts. However, the heterogeneity in suicidal behavior makes it difficult to develop an all-encompassing model of suicide risk or a single prevention and/or intervention pathway [1].

It has been acknowledged that no single strategy stands above others in addressing such a complex multifaceted concern [3]. Instead, there is strong support for a broader public health approach that seeks to tap into synergies between various evidence-based strategies to simultaneously address a range of risk factors for greater impact [2,3,4,5]. This public health approach is necessarily population-based, and prevention-focused, involving multiple avenues and levels of prevention [6]. Within the realm of suicide prevention research, there has been recent attention on suicide prevention strategies that simultaneously implement multiple forms of evidence-based interventions. Evidence related to the effectiveness of such approaches has emerged from high-income countries across the world [7,8,9]. In a systematic review and meta-analysis, Hofstra et al. [10] found that multilevel interventions were more effective than single-level interventions and, further, that effect sizes were significantly higher with the number of levels involved. However, a need for further research on complex interventions and their synergistic potential was emphasized. Despite significant advancements, we still lack evidence on *what works,* and an understanding of *how* evidence-based interventions can be implemented and replicated in real-life settings. An important impeding factor is our lack of understanding of key concepts related to complex interventions in suicide research, which requires further elaborations.

In the context of emerging evidence around complex interventions in suicide research, the objectives of this review are threefold. We seek to (1) explore the breadth of literature on what complex interventions are in public health research; (2) investigate terms and definitions and their implications for suicide research; and (3) summarize key challenges and considerations going forward. The objective is to provide a comprehensive review of an intricate and important topic for the field and for suicide prevention researchers.

## 2. The Complexity in Defining Complex Interventions

A public health intervention can be defined as any action or program intended to deliver a net benefit to the community as well as individuals [11]. To address suicidal behavior, a public health intervention should involve actions targeted at different communities. These can include a range of interventions such as programs (e.g., cognitive behavior therapy), practices (e.g., training of primary care physicians), procedures (e.g., screening for depression), policies (e.g., restriction of access to means), principles (e.g., prevention before treatment), and products (e.g., self-help applications), as well as medication [12]. Characteristics such as the setting of the intervention, the target of change, resources used, and the agent of change can be used to describe different kinds of interventions [13]. McLeroy and colleagues [13] also distinguish between the level—the position in the social ecology (individual, health care system, community) and target—and the entity of focus of interventions. This is to further suggest that they may be implemented at one level but target multiple levels of behaviors/communities/systems. This forms an important premise to understand complex interventions and the intricacies surrounding its definition.

Defining *complex interventions* is somewhat challenging. While the term *intervention* is relatively easy to define as intentional actions to improve health outcomes, the term *complex* is harder to define. One approach could be to define what it is not—that is, a simple intervention. However, this too is not clear-cut. For instance, distributing pamphlets with information on suicide helplines and support services to a community of youth may at first sound like a simple intervention. Even though the messaging may be consistent, different youth may perceive it differently and pamphlets may not reach or be accessible to all kinds of youth if diversity is not taken into consideration. Furthermore, pamphlets may not be adequate and acceptable for a population reliant on digital media and/or populations with low literacy. Implementing this intervention in real-life settings is hardly *simple*. A simple intervention may have unintended ramifications due to inherent complexities in its implementation, the context, or the way it is perceived. Thomas and colleagues [14] argue that interventions as such cannot be simple because when implemented in real life, researchers need to consider one or more aspects of complexity in its design and implementation. Hence, they refer to *intervention complexity* rather than *complex intervention*.

In real life, interventions do not fit into either/or, simple, or complex categories. It has been argued that the way an intervention is defined depends on its characteristics, the research questions involved, and the complexity of analysis and impact [15]. According to this logic, all interventions can be either complex or simple depending on the pragmatic perspective adopted by the researcher to better understand the intervention in question. Characteristics of simplicity and complexity have also been conceived as existing on a spectrum [16].

Despite these difficulties, there have been attempts to define complex interventions. Richards et al. [17] present an overview of definitions based on different characteristics of complexity—the intervention components, implementation, evaluation complexity, and the context. From the perspective of intervention components, complex interventions have been described as comprising multiple components with a unique, complex, and interacting relationship [18]. Anderson et al. [19] argue for the application of a more precise and consistent language to distinguish between conceptually distinct characteristics of complexity. This includes considering the intervention and its characteristics, the varying characteristics of the implementation process, variant properties of the setting or the context, and the variant characteristics of the participant responses. Skivington et al. [20] contend that:

An intervention might be considered complex because of properties of the intervention itself, such as the number of components involved; the range of behaviours targeted; expertise and skills required by those delivering and receiving the intervention; the number of groups, settings, or levels targeted; or the permitted level of flexibility of the intervention or its components (p. 2).

Another view of complexity focuses on the role of complex adaptive systems and not the characteristics of the intervention per se [21]. A complex adaptive system involves an interplay between agents such as professionals, consumers, and organizational systems. The interaction between these agents following simple rules further gives rise to a complex system that is continually adapting to sustain itself [22]. Paradoxically, due to the continually evolving interaction between these agents in the system, it can never be fully resolved or understood [23].

Historically, there has been a tension between developing a better understanding of complex interventions and the perils of over-defining them. Complex interventions are difficult to define considering the *active* and *moving* components of the intervention and their unique ways of interacting with one another [16]. There is fluidity, such that it is difficult to arrive at its exact definition. One approach is to break down the intervention into its constituent components to better understand what it comprises [20]. However, such an approach can lead to “an irretrievable loss of what the complex system used to be” [24] (p. 1561) and an oversimplification of what the intervention does to the system. A complex intervention is hence argued to be more than the sum of its parts [24]. To summarize, the key to defining an intervention is to step back and consider if we are asking simple or complex questions about the intervention.

While the fluidity and complexity of real-world interventions embraces diverse characteristics, it also creates challenges for suicide prevention researchers, particularly around terminology. Frequently the term *complex* is used in a haphazard way to ambiguously to describe interventions. However, it is not always clear (and indeed it is unlikely) whether the same meaning is consistently used and applied across the literature. More specifically, *complexity* is more often used to describe interventions as *complicated*, *difficult to do* and *unclear* which are different concepts [21]. As a result, several terms and definitions are used to denote complex interventions. There is a need for the field to address this confusion and uncertainty around interrelated terms.

## 3. Terms and Definitions

Several interchangeable terms have been used in the literature to connote complex interventions. Some of these commonly found interrelated terms are *multilevel*, *multicomponent*, *systems approach*, *community-based*, *multimodal*, and *integrated* interventions.

A characteristic feature of complex interventions is its *multilevel* approach. *Complex* and *multilevel* are two terms that are often used synonymously to describe interventions with a socio-ecological lens or approach. Such an approach looks at human behavior as influenced by various *levels* within an ecosystem [25]. The approach provides a multilevel conceptualization of the determinants of health and human behavior. Each layer of the ecosystem has multiple actors (such as the individual, healthcare, workplace, school, laws, policies, etc.), which then become targets and/or key actors in the delivery of an intervention. Such interventions aim to create change at different levels—individual and community levels, based on the assumption that the linkage between interventions will create a compounded effect on individual behavior [26]. Hence, such an approach leverages the bidirectionality of relationships between systems. Multilevel interventions have also been defined by Trickett and Beehler [27] as: “… interventions with multiple components designed to affect factors in two or more levels of the local ecology that contribute to wellness and illness, with the goal of effecting changes within and between different levels” (p. 2).

In suicide research, two other approaches have been used [28] to define a *multilevel* approach. The first is the staged traditional prevention approach which comprises three levels: primary (to prevent onset), secondary (to detect and treat), and tertiary (to reduce relapses and recurrence) [29]. The second is a more recent suicide prevention-specific approach, which focuses on the effectiveness of interventions [2,3,4]. Effective interventions are aligned with suicide risk factors and classified into three levels of prevention—universal (for the whole population), selective (aimed at high-risk groups), and indicated (focused on high-risk individuals with history) [30,31]. In this context, a *multilevel* intervention would involve strategies at different levels of prevention, involving the general population or specific sub-groups and individuals.

Similarly, the *systems approach* is based on the understanding that everything is interrelated and interdependent. The *system* can be any cohesive group—a school, community, healthcare setting, family, etc.—which comprises different components or parts. These parts are interdependent, comprise multiple feedback processes and interconnections, and incorporate multiple perspectives [32]. A systemic intervention or one that employs a *systems approach* involves a purposeful action or an intervention to create change in cognizance with the interdependencies of a system [33]. Hence, such an approach involves multiple levels and targets. In the context of suicide prevention, a systems-based approach has often referred to the implementation of *multifaceted* interventions, simultaneously within a region [34].

To add further to the complexity, another commonly used term to denote complexity is *multicomponent*. Public health interventions can vary according to the type and scale of action. Some consist of single strategies and others consist of a combination of strategies or components for population impact on health. As the name suggests, *multicomponent* interventions comprise different components, targeted at the same level or multiple levels of a system. In practice, it is difficult to clearly delineate the *intervention units* or *components* within an intervention [35]. This further makes the definition of multicomponent problematic. A few scholars have attempted to address this challenge by operationalizing the term *components*—those that play a functional role in the theorized change process [36]; those that are theoretically important to the intervention [37]; and active ingredients [38]. Others such as Hawe [36] contend that the functional role of components within an intervention and how they create the intended effect is more important to define than just characteristic features of the component itself. For example, if a component is a suicide awareness media campaign, it is more important to describe how the campaign works and contributes to change rather than what the campaign is, per se.

The term *community-based* is also used to imply complexity and has a wide range of meanings [13]. It often refers to the *setting* in which interventions are implemented. Such interventions may be implemented in a variety of community settings such as schools, health care facilities, neighborhoods, organizations, and other settings. For instance, school-based universal prevention program for suicide could be called a community-*based* intervention. Such interventions may also *target* community change, in creating health community environments through systemic change. For instance, the Zero Suicide Framework [39] aims to reform healthcare settings and hence uses the healthcare community as the *target* of change. Community-based interventions may also *use* communities as a *resource* in terms of building community ownership and partnership. For instance, working with the media to spread awareness about depression and suicide. Closely linked to this idea, is the use of the community as an *agent*. This involves leveraging the existing capacities of communities for change. An example is gatekeeper training with community stakeholders to enhance their capacity to identify those at risk of suicide. Principles of equity, participation, and collaboration to build community capacity and accomplish community-level change is a complex endeavor.

Other less commonly used terms to denote complexity are *multimodal* and *integrated*. These terms carry many connotations. While a multimodal intervention is one that is characterized by different modes of activity or occurrence, integrated interventions refer to linkage and intersectoral collaborations.

**Figure 1:**
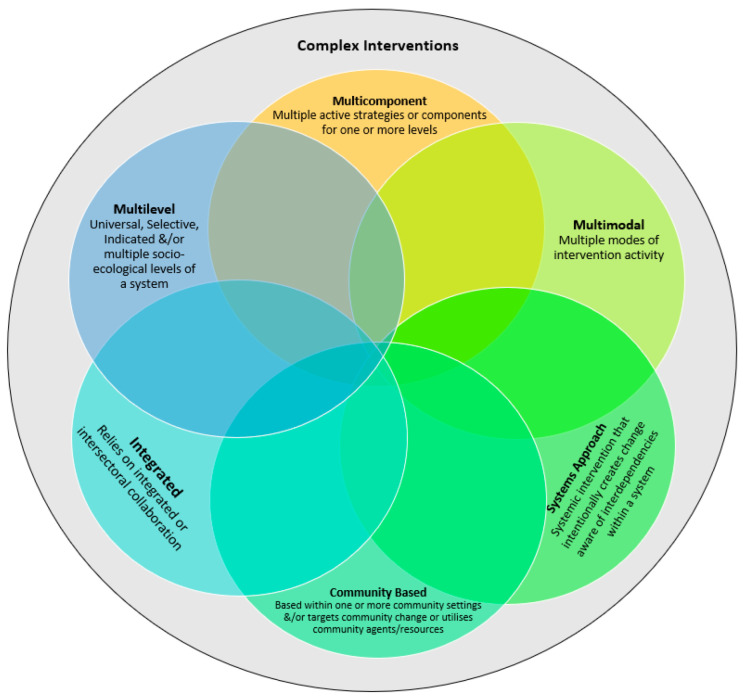
Characteristics of complex interventions.

By their very definition, the terms represent distinct yet overlapping aspects or characteristics of a complex intervention. Some interactions between these terms have been noted in the literature. For instance, the Medical Research Council’s [40] framework for the design and evaluation of complex interventions suggests that multiple components are the building blocks of a complex intervention, which are interacting and impact multiple levels of an ecosystem. Terms such as *multilevel*, *multicomponent*, *multimodal*, *systemic*, *community-based*, *multimodal*, and *integrated* interventions can be understood to be different characteristic types of complex interventions. *Complex intervention* can, hence, be defined as an umbrella term encompassing all these characteristics, among many others as can be seen in Figure 1.

## 4. Challenges and Future Directions

In the context of these commonalities, some important questions emerge—Why is it important to engage in a discussion around semantics? What is the resolution to this complex debate? How does this guide the course of suicide research in the future?

As Ciliska and colleagues [41] note, “Closing the gap from knowledge generation to use in decision-making for practice and policy is conceptually and theoretically hampered by diverse terms and inconsistent definitions of terms” (p. 131). Inconsistent use of language for describing interventions hinders knowledge translation and building an evidence base. The implementation and evaluation of complex interventions in suicide research are novel and hence lack conceptualizations of problems, potential solutions, and a common language. On a practical level, this creates difficulties in extracting and synthesizing information about complex interventions and learning from each other’s work due to variability in the terms and definitions used. Furthermore, language is an important tool to communicate how we think and perceive the world. Reflected in Trickett’s [42] writing, for instance, the use of terms such as *multilevel* is not only reflective of the type of intervention but also the adoption of an approach, a worldview, and the nature of questions posed to better understand the complex problem at hand.

While definitions help in setting boundaries or limits to how reality is perceived, they also tend to oversimplify a very complex reality. Traditionally, researchers have broken down and studied parts of a system (people, intervention, and the outcome) as distinct variables, which come together as a whole to create an effect [22]. For instance, attempts to achieve a consistent terminology around interventions have meant creating a framework including definitions of strategies and techniques, causal mechanisms, modes of delivery, and intended targets [43]. In reality, this picture is not so straightforward; these relationships are complex and unpredictable and cannot be broken down and/or confined within the limits of a definition. Some of these challenges have been documented by [35], attempting to apply the International Classification of Health Interventions (by the World Health Organization) to classify public health interventions. The challenges noted are—consistently identifying separate components within complex interventions; operationalizing the concept of intervention target when there are different kinds of targets during an intervention; coding an intervention component that involves more than one target or action; and standardizing what is being counted.

On a closer examination, this inconsistency is also represented in suicide literature. For example, a literature search on complex interventions in suicide research yielded a variety of terms to connote complex interventions. The most commonly used terms were *community-based intervention*, followed by *multilevel approach*/*intervention* and *suicide prevention program,* each alluding to different aspects of complexity. Interestingly, these terms varied across different publications of the same intervention. For instance, several records were found for the *European Alliance Against Depression* (*EAAD*) and *Optimizing Suicide Prevention Programs and Their Implementation in Europe* (*OSPI*)—a complex suicide prevention intervention alliance in Europe. In one publication the intervention was referred to as a *community-based intervention* [44]; in a later publication, the same intervention was referred to as a *multifaceted*, *community-based action program* [45]. Other terms included a *multilevel approach* [45] and a *complex intervention* [46]. These overlaps were also found across other complex interventions and were indicative of fluidity.

Hence, there is an obvious tension between the need to define a shared understanding and the responsibility to represent the real-life context for what it is. This debate around terms and definitions also creates difficulties in the way evidence-based standards of care are defined. This is summarized by Plsek and Greenhalgh [23] as “the paradox between the need for consistent and evidence-based standards of care and the unique predicament, context, priorities, and choices of the individual” (p. 626). This is reflective of an innate human need to simplify the world into clearly defined categories, which is quite distant from how different phenomena occur. Recently, there has been strong advocacy against reductionist thinking reflected in simplistic research objectives and design to study complex real-life phenomena. Some scholars such as Hawe et al. [24] and Kessler and Glasgow [47] propose the use of pragmatic, transparent, contextual, and complex research designs, which do justice to the complexities of real-world settings. Plsek and Greenhalgh [23] highlight the important contribution of complexity theory in accepting the chaos and unpredictability of the real world. They suggest utilizing multiple approaches to observe, document, and understand patterns within the system which can further aid in arriving at a shared understanding of the system.

Skivington et al. [20] argue that important questions pertaining to the intervention need to be raised and addressed by researchers at key stages of the research process—“How does the intervention interact with its context? What is the underpinning programme theory? How can diverse stakeholder perspectives be included in the research? What are the key uncertainties? How can the intervention be refined? What are the comparative resource and outcome consequences of the intervention?” (p. 1). Furthermore, according to Kessler and Glasgow [47], some practical questions also need to be asked—“What does it cost? How many and what types of people will participate and how do I know this will work in our setting? Will this research generate data likely to result in policy or practice improvement within 3–5 years?” (p. 639).

These questions are important to capture the complexity of how interventions interact with real-world settings. While the literature on complex public health interventions is rich in *descriptions* of complex and challenging interventions, there is little *practical* advice on how these should be implemented. Concurrently, our understanding of complex interventions in suicide research is still quite nascent, complicated further by the lack of agreement and understanding on what complex interventions are and how to define them.

There are myriad advantages of having a common language. It facilitates communication, aids scholarly discourse, and supports transparency. This can also help contribute to a sense of cohesion, community and collaborative problem solving. An important objective of a common language is to develop a scientific basis for understanding characteristics of complex interventions. This can further contribute to the improved implementation and evaluation of complex interventions in real-life settings. However, there are some obvious limitations and unintended consequences of imposing a common conceptual language.

While it may help remove ambiguities, it may also oversimplify concepts. For example, a *component* in a complex intervention may be defined as a constituent part of the intervention—such as an awareness campaign and a means restriction campaign. However, this definition does not comprehensively capture the complexities involved in its implementation on the ground and/or how it relates to the *other* components of the intervention. In theory, having clearly defined components would make the intervention more amenable to systematic measurement. It would also help ascertain the effectiveness of each separate component. However, in practice, this is difficult due to the absence of distinct characteristics and blurred boundaries. As mentioned, interventions and/or actions do not neatly fit within the limits of a definition. For example, a suicide awareness campaign and a means-restriction campaign may be conceptually two separate *components* but may be designed to work in conjunction with each other when implemented in a real-life setting. In such circumstances, it would be important for researchers to have clarity and foresight regarding the actions undertaken while implementing a component and their potential synergies when implemented in a real-world context. The need for simplicity and clarity in how concepts are defined for a generally shared understanding is therefore a major practical limitation to developing a common conceptual language for such complexities. Furthermore, language and culture are intricately woven. This creates concerns around the applicability and acceptability of such a common language across different research environments and contexts. Perhaps, the answer lies in understanding and documenting the operational and functional aspects of concepts. There is a strong need for a transparent means of documenting these complexities and what was done as part of the *intervention* [48,49]. Instead of looking for solutions in clear definitions, it may be more important to explore and examine the nuanced characteristics of interventions.

Some efforts are underway. The Medical Research Council (U.K.) has developed and continuously updated its guidelines on developing and evaluating complex interventions [16,20,40]. These seminal publications offer some guidance and directions for defining complex interventions and identifying factors contributing to complexity. There is an increasing appreciation of the growing complexity of healthcare, which is often reflected in the conduct and use of systematic reviews and meta-analyses for informing healthcare practices. In 2013, the Agency for Healthcare Research and Quality (AHRQ), the Evidence-based Practice Center Program in the U.S.A launched a methods development program around systematic reviews of complex interventions [50]. These efforts have led to the development of practical ways to integrate and accommodate knowledge around complex interventions to further aid better decision making about health care.

In this review, we identified gaps in our understanding and reporting of complex interventions in suicide research. Unless we capture *how* an intervention was conceptualized, implemented, and evaluated, key lessons will be lost such that each intervention effort would involve starting over again to develop this knowledge. Hawe [36] summarizes key lessons for researchers from implementing complex interventions in health research. From an intervention perspective, embracing *complexity* means to accept and acknowledge that the real world is complex and unpredictable. Hence, complexity needs to be embedded into theorizing about how a program and its components *function*. Interventions must be designed considering their relevance and ecological fit without a singular focus on effectiveness. In embracing complexity, asking pragmatic questions about how to evaluate the dynamics of complex interactions, costs involved, and what constitutes fidelity are critical. Apart from a careful consideration of these questions, a clear theory, logic, and rationale underpinning implementation and evaluation needs to be applied. It is important that the limits of models or frameworks along with potential gaps are clearly defined.

From an implementation perspective, representing complex interventions for what they are can help identify and address crucial evidence-practice translation and quality of care gaps in suicide prevention. As a community of suicide researchers, what we need is a systematic approach to understand and document what comprises a complex intervention. This understanding could emerge from existing guidelines and frameworks which have been effectively utilized in public health research. Alternatively, this understanding could also grow out of our experiences of conceptualizing, implementing, and evaluating complex interventions. Such a shared understanding can potentially contribute to quality implementation of best practice care. Other important ways would be to be intentional in the dissemination of research output such that the *intervention* and its practical considerations are clearly described, regardless of the terminology used.

A few limitations should be acknowledged with the current review: First, we did not conduct a systematic literature review. Hence, the literature reported is comprehensive but not exhaustive. Only English language papers were included in the review, due to which papers reported in other languages may have been missed. Considering the challenges and ambiguity surrounding complex interventions, a systematic review would be valuable for providing an exhaustive picture, and acknowledging the implications of inconsistent reporting in peer-reviewed literature.

## 5. Conclusions

To summarize, the more difficult it is to define an intervention, the greater the likelihood that we are dealing with a complex intervention [40]. Complex interventions are important and hold the promise of accounting for real-life complexities. While the benefits are unparalleled [27], there is very little understanding regarding how these interventions interact with and contribute to change within a system [26]. Shifting the focus from what interventions are to how they work in a real-world setting may help create public benefit.

## Data Availability

Not applicable.
